# Clinical and therapeutic course in head variants of linear morphea in adults: a retrospective review

**DOI:** 10.1007/s00403-022-02478-1

**Published:** 2022-12-02

**Authors:** Winnie Fan, Bianca Obiakor, Rebecca Jacobson, Anna Haemel, Jocelyn Gandelman

**Affiliations:** 1 School of Medicine, University of California, San Francisco, San Francisco, CA USA; 2 Department of Dermatology, University of California, San Francisco, San Francisco, CA USA

**Keywords:** Morphea, Parry Romberg syndrome, PRS, *En coup de sabre*, ECDS, Head variant morphea, Complications

## Abstract

Parry Romberg Syndrome (PRS) and *en coup de sabre* (ECDS) are head variants of linear morphea with functional and structural implications. This study describes the clinical course, autoimmune co-morbidities, complications, and treatment of adults with PRS/ECDS at a tertiary referral center. We retrospectively reviewed the records of all 34 adult patients with PRS/ECDS identified through billing code search and seen by dermatologists at our institution between 2015 and 2021. Eight patients (23.5%) had ECDS, 8 (23.5%) had PRS, and 18 (52.9%) had overlap. Twenty-six patients (76.5%) reported ocular, oral, and/or neurologic symptoms, and 8 (23.5%) had concomitant autoimmune/inflammatory conditions. Sixteen patients (47.1%) had a skin biopsy, and 25 (73.5%) had imaging. Forty-six MRIs were obtained, of which 6 (13.0%) reported intracranial findings and 25 (54.3%) reported disease-related connective tissue damage. Twenty-four patients (70.6%) underwent systemic treatment during their disease course per available clinical records. Seventeen patients (70.8%) had improved or stable disease upon treatment completion, with an average duration of 22.2 months. Ten patients (41.7%) reported recurrence of disease following the treatment course. To address changes to facial contour, 6 patients (17.6%) opted for procedural treatments. One patient (16.7%) experienced morphea reactivation following a filler injection performed off-immunosuppression. Compared to findings in children, our study suggests adults with PRS/ECDS are more likely to have oral and ocular complications but experience less severe neurologic symptoms. While systemic treatments appear beneficial in most adult patients with PRS/ECDS, disease may recur following discontinuation.

## Introduction

Morphea is a rare sclerosing skin disorder of unclear pathogenesis affecting both children and adults. Parry Romberg Syndrome (PRS) and *en coup de sabre* (ECDS) are head variants of linear morphea. PRS refers to hemifacial atrophy, whereas ECDS refers to linear sclerotic lesions on the paramedian forehead or frontoparietal scalp; these conditions may overlap [1]. Linear morphea subtypes, including PRS and ECDS, are more common in children [2–7]. Depending on the extent of the disease, PRS and ECDS may involve the dermis, subcutaneous tissue, muscle, or bone, as well as underlying or anatomically related structures [2].

Extracutaneous manifestations (ECM) of head variants of linear morphea include neurologic, ocular, and oral complications [7–11]. However, the literature is incomplete, in part due to variation in clinical assessment, imaging, and other methods of screening for deeper involvement. There is currently no universal diagnostic test for PRS/ECDS and no pathological marker that correlates with disease activity [12–14]. The utility of employing Magnetic Resonance Imaging (MRI) to monitor disease activity and associated ECM has been demonstrated in adults and children with morphea of the trunk and/or extremities [15–18]. However, a correlation between abnormal MRI imaging and associated neurologic ECM has not yet been established [19–23].

For patients with ECDS and PRS, early diagnosis and treatment to halt disease activity are critical to prevent damage and functional impairment [24, 25]. The gold standard for treatment consists of immunosuppressive and immunomodulatory treatments to control active inflammation and prevent further damage [26–30]. A randomized prospective study examining the efficacy of systemic treatment in PRS/ECDS patients has not yet been completed.

The purpose of this retrospective observational study is to expand on the current literature on head variants of linear morphea in adults by describing the clinical course, disease characterization, and management of patients seen at the University of California, San Francisco (UCSF) Department of Dermatology from 2015 to 2021.

## Methods

### Patient selection

**Fig. 1:**
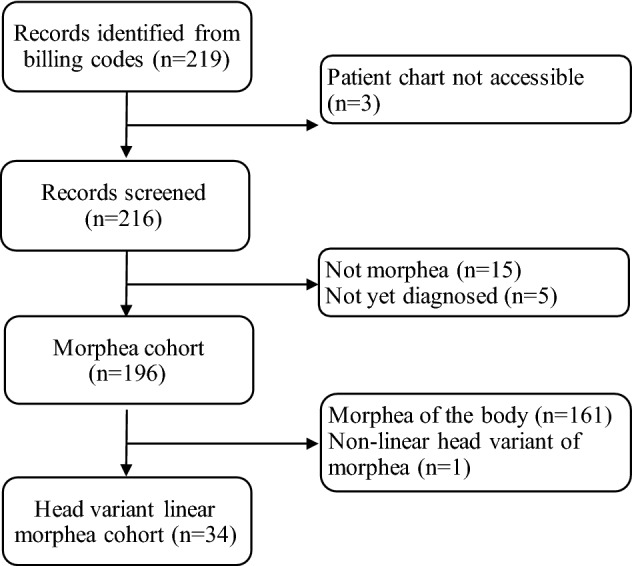
Flowchart depicting selection of the study cohorts

This IRB-approved study is a retrospective chart review of all adult patients ≥ 18 years old with head variants of linear morphea seen at UCSF Department of Dermatology between 01/01/2015 and 03/15/2021. Patients with morphea-related diagnoses were identified via search of relevant ICD-10 billing codes: morphea (L94.0), linear scleroderma/linear morphea/ECDS (L94.1), Parry Romberg (G51.8), and hemifacial atrophy (Q67.4). The electronic health records of patients with at least one of the codes were reviewed by authors W.F. and J.G. to assess for a clinical diagnosis of morphea (Fig. 1). Patient morphea subtypes, including PRS, ECDS, generalized plaque, linear morphea of trunk/extremities, and/or localized plaque, were determined. When chart review led to uncertainties of morphea subtype based on physical examination and history, disease subtype was validated by consensus (AH and JG). Patients with a final clinical diagnosis of ECDS, PRS, or overlap, defined as concurrent ECDS and PRS, per dermatology and/or rheumatology notes were included in the head variant linear morphea cohort.

For the head variant linear morphea cohort, patient charts were reviewed for demographics, age of morphea onset, disease subtype, morphea location, complications, treatment course, and response. Relevant clinical notes and laboratory/pathology/imaging findings were reviewed. Pathology reports of skin biopsies completed for reasons aside from suspected morphea were excluded. The imaging report findings were correlated with those from clinical assessment. The agreement between MRI findings and clinical assessment regarding morphea disease activity and damage was evaluated using percentage of agreement and Cohen’s kappa. Results for continuous variables were summarized as mean ± standard deviation or median, range. Categorical variables were summarized as number of patients (percentage of patients).

### Outcome measurements

We classified the outcomes of treatment as improved, stable, progressive disease, or intolerant of treatment. Definitions were adapted from Arthur et al. 2020 with added clinical assessments in the electronic medical record as one of the criteria for each category [26]. Specifically, improved was defined as absence of new or expanding lesions in addition to one of the following: lesions with decreased erythema or decreased induration, improvement of an associated functional impairment, or clinically assessed as improved. Stable was defined as no change in erythema, induration, size, or number of lesions, or clinically assessed as stable. Progressive was defined as morphea with new or expanding lesions, increased erythema, increased induration, progression of associated functional impairments, or clinically assessed as progressive. Intolerance to treatment was defined as treatment side effects that led to discontinuation of treatment. In this study, treatment response was assessed based on the patient’s response to their most recent systemic treatment during follow-up encounter with their provider. Disease remission was defined as inactive disease for over 6 months or clinician assessment of disease remission. Recurrence was defined as disease activity following a period of remission.

## Results

### Characteristics of the patient cohort

There were 219 patients with morphea-related diagnoses identified through billing code search, with 196 (89.5%) confirmed to have a morphea diagnosis (Fig. 1). Of 196 patients with a confirmed morphea diagnosis, 81 patients (41.3%) had generalized plaque morphea, 53 (27.0%) had localized plaque morphea, and 29 (14.8%) had linear morphea of the trunk and/or extremities. Among patients with generalized plaque morphea, 4 patients (4.9%) had concurrent PRS/ECDS. Among patients with localized plaque morphea, 5 patients (9.4%) had concurrent PRS/ECDS. Most significantly of the 29 patients with linear morphea of the trunk and/or extremities, 5 (17.2%) had concurrent PRS/ECDS.

The patient cohort with head variants of linear morphea consisted of 34 adult patients. Twenty-eight (82.4%) were female, 17 (50.0%) were white, and 22 (64.7%) were non-Hispanic (Table 1). The mean age of the onset of disease was 24.4 ± 15.3 years. Twenty patients (58.8%) had adult-onset of disease, with a mean age of 33.2 ± 13.4 years, whereas the mean age of pediatric onset was 10.9 ± 3.7 years. The average follow-up time was 28.8 ± 34.6 months. Twenty-six patients (76.5%) had follow-up for longer than 3 months.

**Table 1:** Demographic information of head variant linear morphea cohort (*n* = 34)

Item	Number of patients (%)
Sex	
Female	28 (82.4)
Male	6 (17.6)
Other	0 (0.0)
Race	
American Indian or Alaskan native	0 (0.0)
Asian	4 (11.8)
Black or African American	1 (2.9)
Declined	1 (2.9)
More than 1 race	2 (5.9)
Native Hawaiian	0 (0.0)
Other	13 (38.2)
Other Pacific Islander	0 (0.0)
Unknown	0 (0.0)
White or Caucasian	17 (50.0)
Ethnicity	
Declined	1 (2.9)
Hispanic	9 (26.5)
Non-hispanic	22 (64.7)
Unknown	2 (5.9)

Of the 34-patient cohort, 8 (23.5%) had ECDS, 8 (23.5%) had PRS, and 18 (52.9%) had overlapping variants (Table 2). Eleven patients (32.4%) had extra-facial morphea involvement of their trunk/extremities. Not mutually exclusively, 5 patients (14.7%) had linear morphea of other regions of the body, 5 (14.7%) had localized plaque morphea, and 4 (11.8%) had generalized plaque morphea. Five patients (14.7%) had more than one extra-facial morphea type.

**Table 2:** Summary of morphea subtype, extra-facial involvement, and concurrent autoimmune diseases in patients with head variant linear morphea (*n* = 34)

Item	Number of patients (%)*
Subtype	
ECDS only	8 (23.5)
PRS only	8 (23.5)
Overlap	18 (52.9)
Concurrent extrafacial morphea	
Any	11 (32.4)
Generalized plaque	4 (11.8)
Linear morphea of trunk/extremities	5 (14.7)
Localized plaque	5 (14.7)
Concurrent autoimmune disease	
Any	8 (23.5)
Alopecia areata	1 (2.9)
Celiac disease	2 (5.9)
Grave’s disease	2 (5.9)
Juvenile idiopathic arthritis	1 (2.9)
Lupus	2 (5.9)
Pityriasis rubra pilaris	1 (2.9)
Raynaud’s syndrome	3 (8.8)
Sjogren’s syndrome	1 (2.9)
Ulcerative colitis	1 (2.9)

*ECDS* *en coup de sabre*, *PRS* Parry Romberg syndrome

*Values in column do not add up to 100% because some patients had multiple extrafacial morphea types or concurrent autoimmune diseases

Thirty-three patients (97.1%) had a unilateral distribution of morphea lesions, with no lesions of the head crossing the midline. Among patients with ECDS, only 1 (12.5%) had lesions outside of the frontal or parietal areas, in this case occurring at the temporal compartment. All 8 patients with PRS had some oral, perioral, or mental involvement. All 18 patients with overlapping variant had lesions involving the forehead or scalp, with at least one lesion below the forehead (Table 3).

**Table 3:** Summary of morphea lesion distribution in head variant linear morphea cohort (*n* = 34)

Lesion distribution	Number of patients (%)*
All patients (*n* = 34)	
Unilateral	33 (97.1)
Bilateral	0 (0.0)
Midline	1 (2.9)
Patients with ECDS only (*n* = 8)	
Upper face	8 (100.0)
Frontal and/or scalp	8 (100.0)
Temporal	1 (12.5)
Midface	0 (0.0)
Lower face	0 (0.0)
Patients with PRS only (*n* = 8)	
Upper face	2 (25.0)
Frontal and/or scalp	0 (0.0)
Temporal	2 (25.0)
Midface	6 (75.0)
Nasal	3 (37.5)
Malar	5 (62.5)
Lower face	8 (100.0)
Oral and/or peri-oral	7 (87.5)
Mental	6 (75.0)
Overlap (*n* = 18)	
Upper face	18 (100)
Frontal and/or scalp	18 (100)
Temporal	10 (55.6)
Midface	16 (88.9)
Nasal	9 (50.0)
Malar	9 (50.0)
Lower face	12 (66.7)
Oral and/or peri-oral	7 (38.9)
Mental	9 (50.0)

*Values in column do not add up to 100% because some patients had lesions at multiple sites

*ECDS* *en coup de sabre*, *PRS* Parry Romberg syndrome

### Extracutaneous manifestations

In the head variant cohort, 26 patients (76.5%) had oral, ocular, or neurologic clinical symptoms or exam findings related to PRS/ECDS (Table 4). Fifteen patients (44.1%) reported neurologic symptoms, such as headache, migraine, paresthesia, dysphagia, and contralateral extremity numbness. Ipsilateral ophthalmologic symptoms, including enophthalmos, retinal hemorrhage, and vision loss, were observed in 13 patients (38.2%). Ipsilateral oral findings were noted in 16 patients (47.1%), most commonly tongue hemiatrophy and gingival recession. No patients had seizures or increased intraocular pressure. Eight patients (23.5%) were identified as having concomitant autoimmune/inflammatory condition (Table 2).

**Table 4:** Summary of complications associated with head variant linear morphea

Item	Number of patients (%)*
Muscle abnormalities	7 (20.6)
Bony abnormalities	6 (17.6)
Nasal valve cartilage involvement	3 (8.8)
Brain abnormalities	1 (2.9)
Central nervous system symptoms	15 (44.1)
Headaches ipsilateral to morphea lesion	13 (38.2)
Paresthesia at lesion	3 (8.8)
Dysphagia	1 (2.9)
Ipsilateral extremity numbness	1 (2.9)
Seizures	0 (0.0)
Ocular symptoms	13 (38.2)
Brow loss	4 (11.8)
Eyelid	4 (11.8)
Enophthalmos	3 (8.8)
Visual disturbance (blurry vision)	3 (8.8)
Lash loss	2 (5.9)
Ocular motility impairment	2 (5.9)
Visual loss	1 (2.9)
Retinal hemmorhage	1 (2.9)
Chronic iridocyclitis	1 (2.9)
Increase intraocular pressure	0 (0.0)
Visual impairment	0 (0.0)
Oral symptoms	16 (47.1)
Gingival recession	11 (32.4)
Tongue hemiatrophy	10 (29.4)
Temporomandibular joint disorder	4 (11.8)
Gingivitis attributed to morphea	3 (8.8)
Teeth shifting	2 (5.9)
Incisor shifting	1 (2.9)
Incisor implant	1 (2.9)

*Values in column do not add up to 100% because some patients had multiple complications

### Skin biopsies and radiographic imaging

Skin biopsies related to suspected morphea were obtained in 16 patients (47.1%). Twelve patients (75%) had a single biopsy, 3 patients (18.8%) had 2 biopsies, and 1 patient (6.3%) had 3 biopsies, totaling 21 biopsies of suspected morphea. Of the 21 biopsies, 12 (57.1%) had a morphea diagnosis on the pathology report and 5 biopsies (23.8%) were interpreted in the clinical notes as consistent with morphea. Twelve (57.1%) demonstrated both active inflammation and sclerotic changes. Two (9.5%) demonstrated active inflammation without sclerotic changes. One (4.8%) showed sclerotic changes without evidence of active inflammation. Four (19.0%) performed at external facilities lacked clinical data regarding active inflammation and/or sclerotic changes, and 2 (9.5%) reported a line diagnosis unrelated to morphea. A diagnosis of alopecia areata was provided on the pathology reports of 2 patients who were later diagnosed with PRS/ECDS overlap.

Most of the patients underwent imaging. Twenty-five (73.5%) were examined with MRI, computed tomography, and/or plain film, with such imaging obtained for 24 patients (96.0%), 2 (8.0%) and 1 (4.0%), respectively. A total of 46 MRIs were obtained among 24 patients, 11 (45.8%) of whom had follow-up MRIs. Twenty-three patients (95.8%) had MRI reports commenting on intracranial structures, of which 6 (26.1%) had brain abnormalities ipsilateral to their morphea lesions. Most significantly, 1 patient (4.3%) with severe PRS/ECDS overlap had MRIs that showed foci in frontal subcortical white matter, lentiform nucleus, and putamen, which were suggestive of a regional vascular process. This patient’s symptoms included headache, ipsilateral extremity numbness, and dysphagia. Other brain abnormalities observed in 5 MRIs included prominent T2 hyperintensity of the white matter. Of 6 patients with intracranial findings, only 3 (50%) reported CNS symptoms.

Twenty patients (83.3%) had a total of 30 MRIs that examined the connective tissues of the head. Twenty-five MRIs (83.3%) reported morphea-related findings, including subcutaneous tissue, bone, and muscle damage. Thirteen MRIs (43.3%) revealed damage of the underlying structure that was not observed on clinical examination. Among the 11 patients with follow-up MRIs, 3 patients (27.3%) had MRIs that detected progressive tissue loss or active inflammation, which informed management decisions.

The percentage agreement between clinical assessments and MRI findings of soft tissue disease progression was 43.8%, with a Cohen’s kappa of 0.143 (95% confidence interval: − 0.048 to 0.334), suggesting slight agreement. For CNS abnormalities, the percentage of agreement between clinical assessments and MRI findings was 41.7%, with a Cohen’s kappa of − 0.050 (95% confidence interval − 0.303 to 0.203), suggesting no agreement. For connective tissue damage, the percentage of agreement between clinical assessments and MRI findings was 83.3%. Cohen’s kappa cannot be calculated for connective tissue damage because all patients who were referred for MRIs had clinically observed connective tissue damage prior to imaging.

### Treatment

**Fig. 2:**
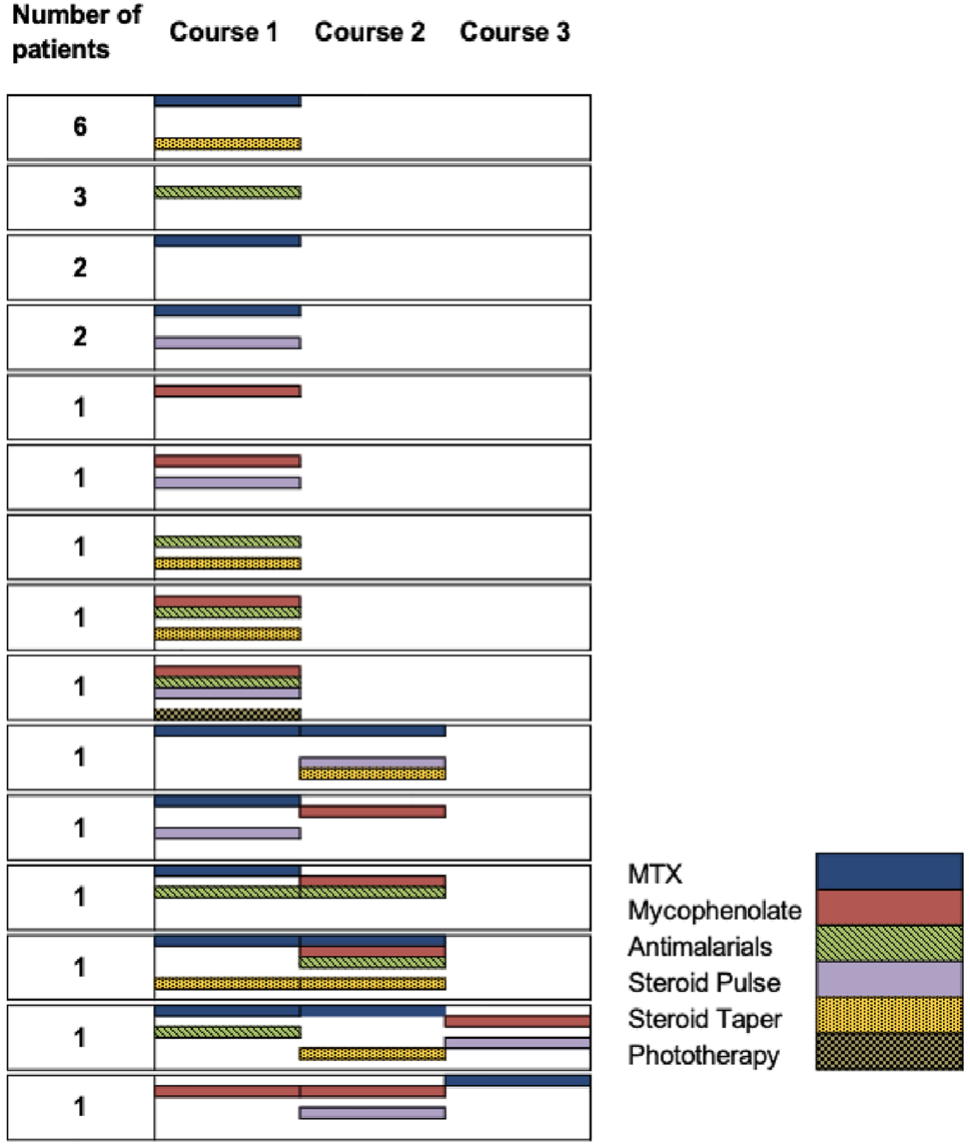
Treatment courses and agents of 24 patients who received systemic treatment. Legend: MTX = Methotrexate; this Figure includes columns for number of patients and shows treatment course 1, course 2, and course 3. For example: six patients had MTX with steroid taper as one course and three patients had antimalarials as one course

Twenty-four patients (70.6%) underwent systemic treatment including methotrexate, mycophenolate, systemic corticosteroids, and/or antimalarials. Three patients (8.8%) only used topicals, 1 (2.9%) only had tissue augmentation with filler, and 6 (17.6%) had no treatments at all during their disease course. The average number of agents used by patients who had systemic treatment was 2.3 agents. Six patients (18%) had more than one course of systemic treatment due to recurrence. Figure 2 provides further details on treatment regimens. Of the 24 patients who had systemic treatment, 16 (66.7%) received methotrexate, 11 (45.8%) received systemic corticosteroid taper, 9 (37.5%) received mycophenolate or analogs, 9 (37.5%) received antimalarials, 8 (33.3%) received systemic corticosteroid pulse, and 1 (4.2%) received phototherapy. Nine patients (37.5%) experienced side effects. Four patients (25.0%) on methotrexate had side effects: mouth sores, abdominal discomfort, hair loss, and elevated liver function tests (each occurring in 1 patient). Three patients (33.3%) on antimalarials had side effects: 2 patients had cutaneous drug eruptions and 1 had malaise. Two patients (18.2%) had side effects on a systemic steroid taper: 1 had face swelling and 1 had anxiety. One patient on mycophenolate had abdominal discomfort.

The average time on treatment was 22.2 months ± 18.8 months. The median time from onset of disease to treatment was 2.5 years, ranging from 0 to 25 years. Of the 24 patients who underwent systemic treatment, 17 (70.8%) had improved or stable disease upon completion of any systemic treatment, 3 (12.5%) had progressive disease, and 4 (16.7%) were intolerant of the initial treatment agent and discontinued systemic treatment. Specifically, 3 patients who experienced side effects from antimalarials and 1 patient from methotrexate proceeded to stop all systemic treatment. Ten patients (41.7%) had reactivation of disease after completion of systemic treatment.

Among patients who received MTX (*n* = 16), 11 patients (68.8%) had improved or stable disease upon completion of treatment, 1 patient (6.3%) had progressive disease, and 4 patients (25.0%) did not tolerate MTX. Of the 4 patients who did not tolerate treatment, 1 patient (6.3%) experienced improvement with a first course of MTX treatment but was intolerant of the agent when reintroduced it for a recurrence. Among patients who received mycophenolate (*n* = 9), 7 (77.8%) had improved or stable disease upon completion of treatment and 2 patients (22.2%) were intolerant of treatment.

Of all 34 patients, 25 (73.5%) patients were stable or in remission at their most recent clinic visit. Eleven patients (32.4%) were stable on medication. Fifteen patients (44.1%) had recurrence of disease activity over the time period documented in their clinical record, with time to recurrence from remission ranging from 8 to 120 months.

To address facial contour changes caused by PRS/ECDS, 6 patients opted for autologous fat transfer, commercial filler, and/or infraorbital implant. One patient (16.7%), who was off immunosuppression, was found to have reactivation of morphea at their 6 month follow-up after a synthetic filler injection.

## Discussion

To the best of our knowledge, this study describes the first cohort of all adult patients with ECDS and/or PRS. Head variants of linear morphea in adult patients are rare. In pediatric studies, head variants of linear morphea accounted for 11.1–18.4% (ECDS 3–17.6% and PRS 0–8%) of patients with morphea, whereas in adult studies, patients with ECDS accounted for 2.4–4.4% and PRS accounted for 0.3–1% of patients with morphea [31]. Research characterizing the clinical and treatment course of PRS/ECDS in adults is important to optimize diagnosis, evaluation, and management of the disease in this age group.

Our study population was predominantly female and white, similar to previous studies [32, 33]. The average age of onset in our cohort was older than that of prior studies of patients with PRS and/or ECDS; over half of our patient population’s disease onset occurred after 20 years of age [1, 34]. A portion of the patients in this study (23.5%) had additional autoimmune or inflammatory conditions beyond morphea, consistent with findings previously reported in adults [33].

In our morphea cohort, the head was as commonly affected as the trunk and/or extremities in patients with linear morphea. This contrasts with two studies of pediatric patients, which found that linear lesions were more common on the trunk and/or extremities (54% and 77.6%) [3, 35]. Interestingly, another study of body site morphea distribution in pediatric patients found that even though the trunk was the most commonly involved region in patients with linear morphea, after adjusting for relative body surface area, the head and neck were more commonly affected than the trunk [36].

More than half of our patients had concomitant PRS and ECDS, affirming the concept of the two diseases existing on a spectrum [37]. These patients generally had vertical lesions in the frontoparietal area with atrophy of the maxillary or perioral regions. As expected, there were a similar number of patients with right sided and left sided lesions, with unilateral involvement being typical [1]. Though this study did not aim to address the etiology of head variant linear morphea, prevalent unilateral distribution may be consistent with speculations that aberrant nerve development and/or inflammation of the nervous system are involved [38, 39].

This study is distinctive in demonstrating that adult patients with PRS/ECDS had functional impairments and ECMs that were different than previously reported in pediatric populations. A significant portion of adult patients had oral and ocular ECMs of the unilateral face. Gingival involvement was a common finding as well. The finding of gingival involvement had only been previously reported in one pediatric and two adult case studies [40–42]. Gingival diseases such as periodontal disease and gingival recession are common in adults in the US [43]. The inflammatory process of PRS/ECDS may accelerate gingival damage from aging in adult patients, resulting in focal disease-related gingival recession, which when progressive may lead to exposed tooth roots.

No patients in this adult cohort experienced seizures, distinct from pediatric studies. For instance, in an international study of 113 pediatric patients with PRS/ECDS, 8.0% experienced seizures, while in a retrospective study, 50.0% of 12 pediatric patients had seizures [3, 7]. Severe neurological complications remained rare among our adult cohort, with most patients with CNS symptoms reporting headaches. In contrast, in pediatric studies, neurological abnormalities and symptoms associated with head variant linear morphea have been widely reported [20, 21, 23, 44]. In one observational study, 19% of the pediatric patients with head variant linear morphea had neurologic involvement detected by MRIs [44]. In contrast, intracranial involvement on brain MRI was uncommon in the study population, and clinical findings of CNS abnormalities correlated poorly with MRI findings. For patients with MRI-detected brain abnormalities in this study, incidental findings unrelated to morphea cannot be ruled out. The small number of adult patients with severe neurologic abnormalities was expected. It was reported that adults tend to experience milder functional impairment and less deep involvement of morphea lesions when compared to children [45]. Moreover, the onset of PRS/ECDS and related inflammatory processes in this adult cohort may have begun after the window of cerebral vulnerability had already passed, making CNS involvement less likely [46].

A study in an adult and pediatric cohort had indicated that MRI could be a useful adjunct to assessing morphea activity and damage [16]. In our study, MRIs detected 83.3% of disease damage observed in clinical assessment. In 3 patients from our study, disease activity was detected based on serial MRIs. As such, MRI images at initial evaluation could be helpful in detecting underlying tissue involvement as well as measuring progression by establishing a baseline for subsequent comparison. It has also been postulated that skin biopsy may assist in identifying severe inflammation and sclerosis in morphea patients [47]. More severe inflammation has been associated with greater functional impairment in one single center, retrospective study, but another study suggested that histopathologic features of morphea can be variable and not of prognostic value [12, 48]. In our study, 61.9% of skin biopsies performed revealed active inflammation and/or sclerosis, helping to confirm morphea in patients with high clinical suspicion for the condition. However, 2 patients ultimately diagnosed with PRS/ECDS overlap had a discordant histopathologic diagnosis of alopecia areata, highlighting matters of test sensitivity and the importance of clinicopathologic correlation.

Treatment of morphea is aimed at halting disease activity and preventing damage. Recommendations for the use of MTX are based on a randomized, double-blind, placebo-controlled clinical trial in a pediatric population comparing the effect of MTX with prednisone to prednisone alone [30]. A recent study investigated treatment with MTX in patients with active ECDS and found response in patients roughly 2 months after the initiation of therapy [34]. In agreement with these findings, over 60% of patients who underwent MTX and/or mycophenolate treatment had improved or stable disease. While systemic treatment showed promise in treating PRS/ECDS, current data were insufficient for determining efficacy. Four patients who experienced side effects stopped systemic treatment entirely, emphasizing the importance of fine-tuning therapeutic strategies to allow safe and effective long-term and/or intermittent treatment.

Similar to prior findings, a significant portion (41.7%) of patients who underwent systemic treatment ultimately had recurrence of disease activity [49]. Of note, one patient experienced reactivation after procedural treatment. It has been suspected that trauma may trigger morphea activation. Hence, several studies have advised that morphea should be quiescent prior to procedural treatments such as fat transfer [50–52].

This single center study is limited by its retrospective nature. Though this was a relatively large cohort of adult patients with EDCS/PRS, the patient numbers were still small; thus, statistical analyses of findings were limited. Not all patients underwent the same clinical testing and evaluation, yet because PRS and ECDS are rare diseases, prospective studies and randomized trials can be difficult to achieve. During the study period, the Localized Scleroderma Cutaneous Assessment Tool (LoSCAT) was not utilized during routine care at our institution, leaving room for inter-observer variability [53]. Three-dimensional photography has been proposed as an additional tool for monitoring patients with head variants of linear morphea and may provide an additional strategy for objective assessment of disease course [54].

## Conclusion

This analysis of 34 adult patients with PRS/ECDS at a tertiary referral center focuses on clinical course of the condition in adults. Key findings include the presence of concurrent autoimmune/inflammatory conditions in over 20% of patients; while local structural complications were common, neurologic complications were less severe than those previously reported in children. To halt morphea progression, over 70% of patients in this study received systemic treatment during the study period. While systemic treatment was effective in most patients (71% stable or improved), relapses of disease activity were not uncommon, with 18% of patients requiring more than one treatment course over the study period. Both clinical follow-up by a multidisciplinary team (dermatology, rheumatology, ophthalmology, dentistry/oral medicine, radiology, and neurology as needed) and patient education on self-monitoring for morphea activity are recommended for optimal long-term outcomes. Further research and randomized controlled studies in head variant linear morphea are needed, particularly due to the chronic and relapsing course that may occur in a substantial proportion of adults.

## Data Availability

De-identified data can be made available upon request on a case by case basis.
